# Deterministic domain wall rotation in a strain mediated FeGaB/PMN-PT asymmetrical ring structure for manipulating trapped magnetic nanoparticles in a fluidic environment

**DOI:** 10.1039/d3ra00150d

**Published:** 2023-01-18

**Authors:** Pankaj Pathak, Vinit Kumar Yadav, Dhiman Mallick

**Affiliations:** a Department of Electrical Engineering, Indian Institute of Technology Delhi New Delhi-110016 India dhiman.mallick@ee.iitd.ac.in

## Abstract

The manipulation of domain walls (DWs) in strain-mediated magnetoelectric (ME) heterostructures has attracted much attention recently, with potential applications in precise and location-specific manipulation of magnetic nanoparticles (MNPs). However, the manipulation ability in these structures is restricted to magnetostrictive circular ring structures only, where the required onion state is metastable, less thermally stable, and cannot be obtained easily. This work investigates the highly shape anisotropic FeGaB magnetostrictive elliptical ring structures of different aspect ratios and trackwidths on the PMN-PT piezoelectric substrate to manipulate fluid-borne MNPs using active control of DWs. The proposed model utilizes the attribute that the required onion state in a magnetostrictive elliptical ring is thermally stable and easily obtained compared to magnetostrictive circular ring structures. By varying the trackwidth of elliptical rings, nucleated DWs are rotated at different angles to capture and transport fluid-borne MNPs. Up to a critical trackwidth, DW rotation is predicted by dominant stress anisotropy energy that leads the rotation of DWs and attached MNPs toward the dominant tensile strain direction of PMN-PT with reversibility. Increasing the trackwidth beyond the critical trackwidth caused a complete 90° rotation of DWs and attached MNPs without reversibility and is given by dominant shape anisotropy energy. The fundamental relationship of capture probability with the size and velocity of injected MNPs is also demonstrated. The nucleation and rotation of DWs are predicated using the coupled elastodynamic and electrostatic Finite Difference Method (FDM) micromagnetic model. Dynamics of MNP capture and rotation are envisaged using an analytical model.

## Abstract 2

The manipulation of domain walls (DWs) in strain-mediated magnetoelectric (ME) heterostructures has attracted much attention recently, with potential applications in precise and location-specific manipulation of magnetic nanoparticles (MNPs).

## Graphical abstract

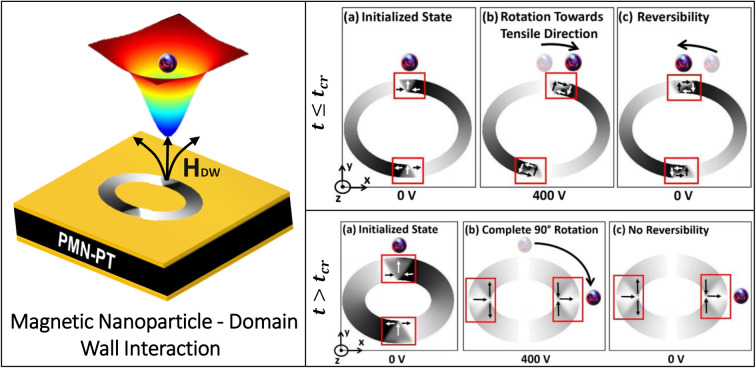

## 1 Introduction

Manipulation of magnetic nanoparticles (MNPs)^1–3^ in a fluidic environment with the precision in the limit of a single cell size is essential for location-specific analysis in various lab-on-a-chip applications. These applications include diverse functionalities in nanobiotechnology,^4,5^ nanochemistry,^6,7^ nanomedicine^5,8^*etc.* Although several techniques have been developed previously to manipulate MNPs at the nano-scale, one of the most successful attempts in this direction is reported by controlling the magnetic domain walls (DWs) in nanomagnetic structures, where highly localized magnetic energy density and its gradient can couple to the MNPs.^9,10^ In preceding studies, conventional methods incorporating external magnetic field^11–14^ and current^15–17^ are investigated widely to control magnetic domains and their DWs. However, these methods are energy inefficient, spatially inaccurate and fall short to perceive the required manipulation. Lately, successful efforts toward this direction are reported using strain-mediated magnetoelectrics (MEs).^18–21^ A ME heterostructure constitutes of magnetostrictive and piezoelectric order parameters.^22^ An applied voltage across the piezoelectric material instigates a strain at the heterostructure interface, altering the magnetization in magnetostrictive material due to the Villari effect.^22,23^

Although previous research suggests that the control and manipulation of the magnetic DWs using strain mediated MEs provides an ultra-low energy route for MNPs manipulation, still this manipulation ability in strain mediated MEs with magnetostrictive/piezoelectric heterostructure is restricted to magnetostrictive circular ring structures only. These circular ring elements show two distinct states: (i) Onion state, in which two semi-circular domains are separated by two DWs and (ii) Vortex state, in which magnetization is circularly oriented either clockwise or anticlockwise.^19,24^ Out of these two states, only the onion state is used to manipulate MNPs due to the high magnetic energy density and its gradient compared to the Vortex state.^9,10,24^ However, the Onion state obtained using a magnetostrictive circular ring is metastable^25–27^ and can not be obtained easily. Also, the obtained onion state is less thermally stable.^28,29^ The thermal stability of DWs is defined as the ability of DW to resist the action of external temperature and to maintain its magnetization properties. Despite successful MNP manipulation, the lower thermal stability of the circular ring can restrict their application for critical biotechnology applications, such as photo-thermal therapy,^30^ ultrasound hyperthermia treatment,^31^ laser-induced hyperthermia^32^*etc.* This is because, in such applications, heated MNPs (>47 °C) are manipulated to reach a target region.^33^ Once captured, heated MNPs can distort the magnetization properties of DWs in the magnetostrictive circular ring structure, which could hinder their manipulation from reaching the desired target location. Also, the additional rotation of the DWs in magnetostrictive circular ring beyond in-plane (IP) 45° requires strains in multiple angles, which is achieved using a multielectrode system,^34–36^ turning the clocking mechanism as well as the fabrication steps complex.

Han *et al.*^28^ demonstrated that the magnetostrictive elliptical ring structures can easily generate the onion state as their shape anisotropy is larger. Also, thermal stability of an onion state in the elliptical ring improves as compared to the magnetostrictive circular ring elements.^28,29^ Although preceding research attempts using elliptical ring structures have focused on memory and logic applications only,^37^ their high thermal stability can provide more comprehensive benefits to critical biotechnology applications related to hyperthermia therapy.^30–32^ Apart from that, additional rotation of DWs beyond IP 45° can be achieved with a simple two-electrode system utilizing shape anisotropy of the elliptical ring. However, magnetostrictive elliptical ring structure has not been explored so far to replace the magnetostrictive circular ring structure in strain mediated MEs for MNPs manipulation, which could possibly make particle manipulation easier and more energy-efficient.

**Fig. 1:**
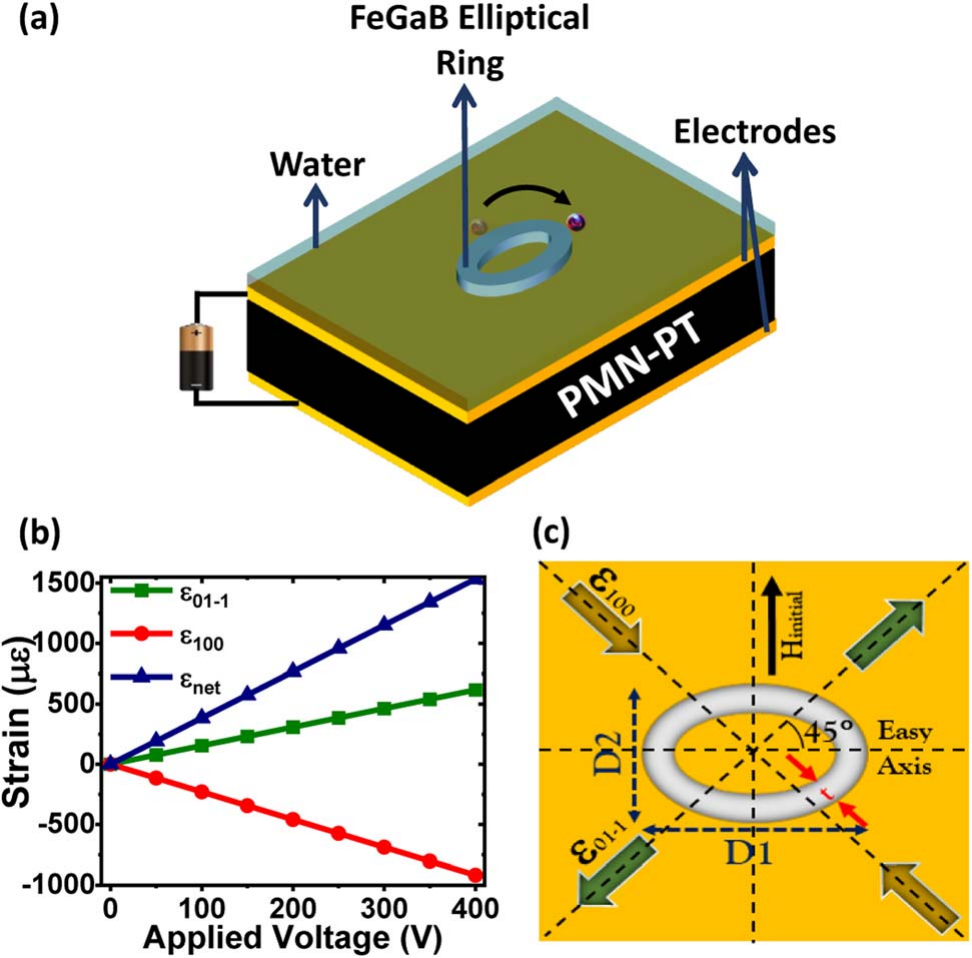
(a) Schematic of prototype geometry investigated in our model (not to scale), (b) strain response of PMN-PT against applied voltage and (c) top view of simulated geometry. Top view of the schematic illustrates typical strain profile at applied voltage. Major axis of the elliptical ring is 45° (clockwise) relative to the tensile strain (*ε*_01−1_) direction of PMN-PT. Initialized magnetic field is applied along the minor axis.

Considering that, we have investigated the manipulation of MNPs in a fluidic environment using elliptical ring-shaped material on strain mediated MEs. For that purpose, elliptical rings of FeGaB on a 0.5 mm thick single-crystal PMN-PT substrate are simulated. The outer diameter (*D*1) of the elliptical ring is fixed at 1 μm, while trackwidth (*t*) and outer diameter along the minor axis (*D*2) are varied. The magnetostrictive material is chosen as FeGaB due to its reasonably large piezomagnetic co-efficient compared to other well-known magnetostrictive materials such as Ni, FeGa, Co *etc.*^22,38^ Also, its lower Gilbert damping coefficient (*α*) reduces power consumption and improves thermal stability.^39^ The PMN-PT with spontaneous polarization along 〈111〉 direction is used as a piezoelectric substrate in our model. The reason for choice of such substrate is that their 〈011〉 cut shows large IP anisotropic strain upon applying a voltage.^40^ The strain profile of PMN-PT as a function of applied voltage is considered linear, as shown in Fig. 1(b). Also, it is assumed that the strain becomes zero upon removal of voltage. Initially, the onion state containing DWs in FeGaB elliptical rings is created. By applying an external voltage across the PMN-PT substrate, these DWs are rotated at different angles depending on the elliptical ring dimension. Subsequently, DW reversibility is examined after removing an external voltage. The DW formation, rotation and reversibility are predicted by Landau–Lifshitz–Gilbert (LLG) equation coupled with elastodynamics using the micromagnetic simulation platform MuMax3.^41^ Next, we magnetostatically coupled the fluid borne MNPs to DWs. Once an external voltage is applied, MNPs motion is observed at different angles depending on the elliptical ring dimension. Using an analytical model, the transport dynamics of MNPs is studied. Also, an analytical model is used to calculate the coupling and drag force between DW and MNPs.

## 2 Theoretical modelling framework

### 2.1 Modeling for domain wall rotation

Our model for controlling the DWs consist of elliptical nano-magnetic rings of FeGaB on PMN-PT substrate, as shown in Fig. 1(a). Our model uses external voltage to instigate the strain in the piezoelectric substrate and assumes 100% strain transfer to magnetostrictive material. The material parameters to model the nano-magnetic rings are at room temperature (300 K). Any thermal fluctuation or noise is neglected as the difference between magnetization dynamics at 0 K and raised temperature is insignificant.^42^ Landau–Lifshitz–Gilbert (LLG) relation (eqn (1)) is numerically solved using MuMax3 (ref. 41) to study the magnetization dynamics of the magnetostrictive elements.1
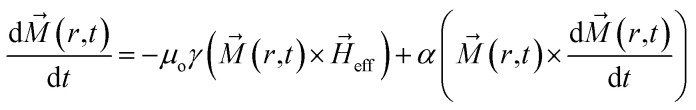
where *μ*_o_, *γ* and *α* are the free space permeability, Gilbert gyromagnetic ratio and Gilbert damping constant respectively. *M⃑*(*r*,*t*) is time and space dependent magnetization vector. As adding boron (B) into FeGa produces a pseudo-amorphous sample, the magnetocrystalline anisotropy (MCA) of FeGaB is considered 0.^43^ Due to this exchange (*H⃑*_ex_) and demagnetization field (*H⃑*_d_) are dominant contribution of effective field (*H⃑*_eff_) when an external voltage is absent. Eqn (2) is used to calculate *H⃑*_ex_2
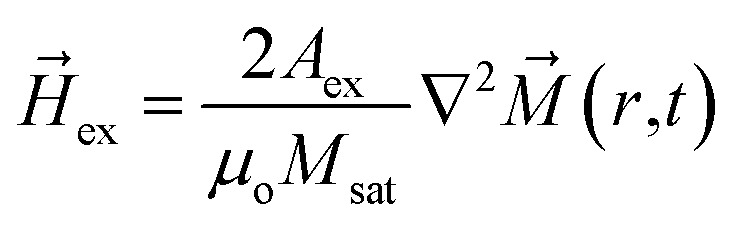
where *A*_ex_ and *M*_sat_ are the exchange stiffness coefficient and saturation magnetization of FeGaB respectively. *H⃑*_d_ is given by

3 *H⃑*_d_ = −∇*ϕ*

where *ϕ* represents magnetic potential and satisfies Poisson equation

4 ∇^2^*ϕ* = *M*_sat_∇·*M⃑*(*r*,*t*)

By applying an external voltage to the piezoelectric substrate, additional stress anisotropy energy (*H⃑*_s_) is produced. *H⃑*_s_ is given by5
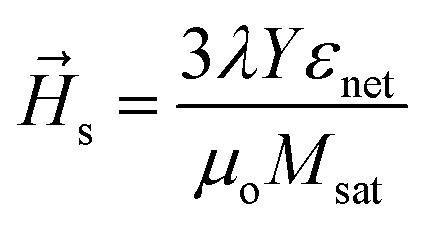
where *λ* and *Y* are magnetostrictive co-efficient and Young's modulus of FeGaB respectively. *ε*_net_ is voltage induced biaxial strain transferred to magnetostrictive material and defined as difference between the strain generated along tensile [01−1] and compressive strain [100] direction *i.e.*

6 *ε*_net_ = *ε*_[01−1]_ − *ε*_[100]_

With 
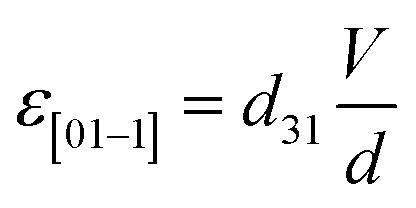
 and 
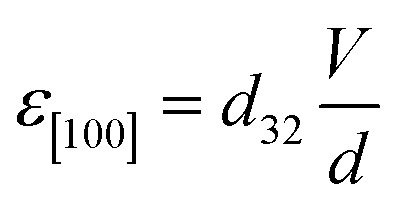
 for which eqn (5) reduces to7
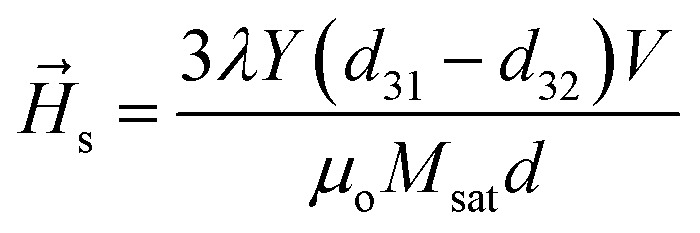
where *d*_31_, *d*_32_ are the piezoelectric coefficient, *V* is an external applied voltage and *d* is thickness of PMN-PT. Here, the piezoelectric coefficients *d*_31_ and *d*_32_ are 771 pm V^−1^ and −1147 pm V^−1^ respectively.^44,45^ The simulated elliptical ring's outer diameter along the major axis (*D*1) is 1 μm for all cases. The trackwidth (*t*) and outer diameter along the minor axis (*D*2) are varied. Three elliptical rings with aspect ratios (AR-*D*1 : *D*2) 1.1 : 1, 1.3 : 1 and 1.5 : 1 with varied trackwidth on a 0.5 mm thick single-crystal PMN-PT substrate are simulated. The thickness of each magnetic ring is 30 nm. As shown in Fig. 1(c) major axis of each magnetic ring is 45° (clockwise) relative to the tensile strain [01−1] direction of PMN-PT. The cell size of the geometries mentioned above is 1 × 1 × 1 nm^3^ with the material parameters for FeGaB as given in Table 1.

**Table 1:** Material parameters for FeGaB^22,46^

Material parameter (unit)	Assigned value
Saturation magnetization (A m^−1^)	9.78 × 10^5^
Exchange stiffness (J m^−1^)	1 × 10^−11^
Magnetostrictive coefficient (ppm)	65
Young's modulus (GPa)	62.4
Gilbert damping constant	0.02

### 2.2 Modeling of magnetic nanoparticle manipulation

For predicting the rotation of magnetic nanoparticles (MNPs) in a fluid environment using DW rotation, spherical iron oxide-based Fe_3_O_4_ nanoparticle is considered, which has a density 5000 kg m^−3^, volume susceptibility 800 kA m^−1^ T^−1^ and saturation magnetization 4.78 × 10^5^ A m^−1^.^46^ Fe_3_O_4_ is considered as it is biocompatible, chemically stable, non-toxic and inexpensive.^47–49^ In this paper, the term magnetic nanoparticle (MNP) is often used interchangeably with the nanoparticle. It is assumed that fluid is non-magnetic water with density 1000 kg m^−3^, permeability 4π × 10^−7^ H m^−1^ and viscosity 0.001 kg m^−1^ s^−1^.^48^

Several forces govern the transport dynamics of MNPs due to DW rotation in the magnetic ring, which includes force due to DW stray field, viscous drag force due to fluid viscosity, surfactant force, gravitational, inertia force on nanoparticle *etc.* For most applications, force due to DW stray field and viscous drag force are the dominant contributions of total force and other forces can be ignored.^19,50^ In this paper, the transport dynamics of a MNP is considered in the non-flow regime, *i.e.* velocity of the fluid is zero. Newton's second law equation is solved to study the nanoparticle motion, as given in eqn (8)

8 *F⃑* = *F⃑*_dw_ + *F⃑*_d_ = *m*_p_*

<svg xmlns="http://www.w3.org/2000/svg" version="1.0" width="14.727273pt" height="16.000000pt" viewBox="0 0 14.727273 16.000000" preserveAspectRatio="xMidYMid meet"><metadata>
Created by potrace 1.16, written by Peter Selinger 2001-2019
</metadata><g transform="translate(1.000000,15.000000) scale(0.015909,-0.015909)" fill="currentColor" stroke="none"><path d="M560 840 l0 -40 -200 0 -200 0 0 -40 0 -40 200 0 200 0 0 -40 0 -40 40 0 40 0 0 40 0 40 40 0 40 0 0 40 0 40 -40 0 -40 0 0 40 0 40 -40 0 -40 0 0 -40z M240 520 l0 -40 -40 0 -40 0 0 -80 0 -80 -40 0 -40 0 0 -120 0 -120 40 0 40 0 0 -40 0 -40 120 0 120 0 0 40 0 40 80 0 80 0 0 -40 0 -40 40 0 40 0 0 40 0 40 40 0 40 0 0 40 0 40 -40 0 -40 0 0 -40 0 -40 -40 0 -40 0 0 160 0 160 40 0 40 0 0 80 0 80 -40 0 -40 0 0 -40 0 -40 -40 0 -40 0 0 40 0 40 -120 0 -120 0 0 -40z m240 -160 l0 -120 -40 0 -40 0 0 -80 0 -80 -120 0 -120 0 0 120 0 120 40 0 40 0 0 80 0 80 120 0 120 0 0 -120z"/></g></svg>

*_p_

Here effective force (*F⃑*) is vector sum of force due to DW stray field (*F⃑*_dw_) and fluid viscosity (*F⃑*_d_) on nanoparticle. *m*_p_ and *α⃗*_p_ are mass and acceleration of nanoparticle respectively. As 
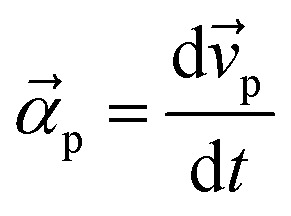
, eqn (8) reduces to9
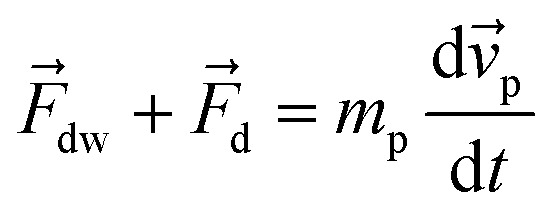
where *v⃑*_p_ is nanoparticle velocity. In eqn (9)*F⃑*_dw_ is given by

10 *F⃑*_dw_ = *μ*_o_*V*_p_[(*M⃑*_p_ − *M⃑*_f_)·∇)]*H⃑*_dw_

where *μ*_o_, *V*_p_ and *M⃑*_p_ are free space permeability, nanoparticle volume and magnetization respectively. *H⃑*_dw_ is magnetic field due to DW stray field at the center of nanoparticle and *M⃑*_f_ is fluid magnetization. As already discussed fluid is non-magnetic (*M⃑*_f_ = 0), eqn (10) can be written as

11 *F⃑*_dw_ = *μ*_o_*V*_p_(*M⃑*_p_·∇)*H⃑*_dw_

We have considered linear magnetization for nanoparticle below saturation *i.e.*12
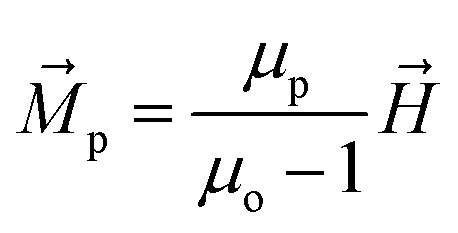
where *μ*_p_ is permeability of nanoparticle and *H⃑* = *H⃑*_dw_ − *H⃑*_dp_, where 
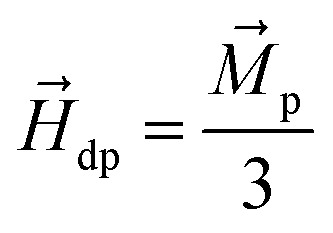
 is self-demagnetization field of nanoparticle.^50^

Using Stokes' law *F⃑*_d_ is given by

13 *F⃑*_d_ = −6π*ηr*_p_(*v⃑*_p_ − *v⃑*_f_)

where *η* and *v⃑*_f_ are the fluid viscosity and velocity respectively. *r*_p_ is nanoparticle radius. As nanoparticle is in non-flow regime (*v⃑*_f_ = 0), eqn (13) reduces to

14 *F⃑*_d_ = −6π*ηr*_p_*v⃑*_p_

The negative sign in eqn (14) indicates that *F⃑*_d_ acts opposite to *F⃑*_dw_. Because Brownian motion can influence the coupling of nanoparticle to DWs when the nanoparticle is sufficiently small, following criteria is used to estimate the critical radius (*r*_c_) of the nanoparticle^51^15
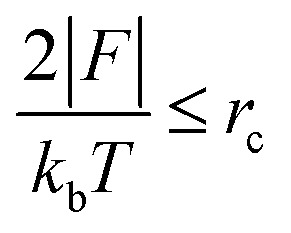
where |*F*|, *k*_b_ and *T* are the magnitude of effective force, Boltzmann's constant and temperature respectively. Eqn (8) is valid only when *r*_p_ ≥ *r*_c_.^51^ For *r*_p_ < *r*_c_, advection–diffusion equation^52^ is used rather than Newton's equation which is beyond the scope of current work. For Fe_3_O_4_ in water critical radius is 80 nm at the room temperature.^50^ In our work *r*_p_ = 100 nm, 200 nm and 300 nm; thus, Brownian motion is neglected. We assume that the MNP is injected with the average velocity (*v⃑*_p_) of 0.1 mm s^−1^, for which force due to fluid viscosity or drag force (*F⃑*_d_) calculated is 0.1884 pN, 0.3768 pN and 0.5652 pN for *r*_p_ = 100 nm, 200 nm and 300 nm respectively, using eqn (14).

To couple the nanoparticle to DW, |*F⃑*_dw_| > |*F⃑*_d_|. This condition predicts that as long as *F⃑*_dw_ surpasses *F⃑*_d_ during DW rotation, nanoparticles bind to the DW and track their location. This confirms the successful nanoparticle rotation. If |*F⃑*_d_| overcomes |*F⃑*_dw_|, the nanoparticle does not bind to the DW.

## 3 Results and discussion

### 3.1 Domain wall initialization

**Fig. 2:**
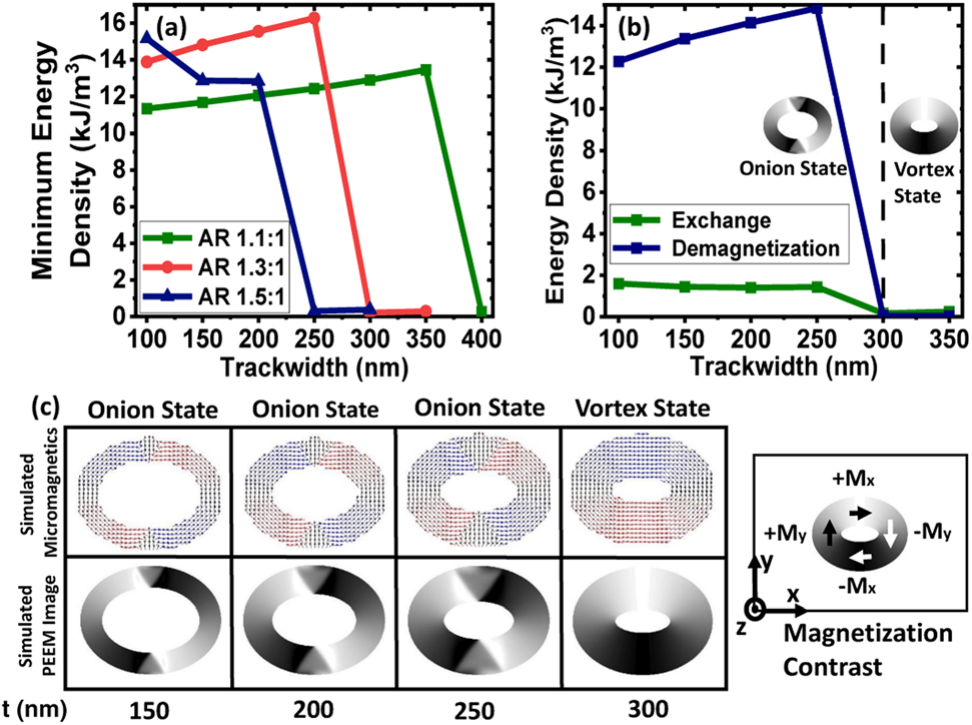
Domain wall initialization for simulated elliptical rings with different aspect ratios. (a) Minimum energy density for elliptical rings with AR 1.1 : 1, 1.3 : 1 and 1.5 : 1 as a function of trackwidth. The nature of the minimum energy density state is observed due to competition between the exchange and demagnetization energies, which are the dominant contribution to the total energy in the absence of an external voltage (b) exchange and demagnetization energy variation against trackwidth for elliptical rings with AR 1.3 : 1 and (c) corresponding simulated micromagnetics and PEEM images. Up to 250 nm trackwidth, the required demagnetization energy to rotate the magnetization 180° is high, producing an onion state with transverse DWs having a non-negative minimum energy density. For trackwidths greater than 250 nm, the required demagnetization energy to rotate the magnetization 180° is low, producing a vortex state with almost negligible minimum energy density. To illustrate magnetization orientation in simulated PEEM images, magnetization contrast is illustrated.

Initially, an external magnetic field is applied along the minor axis of the magnetic rings and subsequently removed after saturation. At the remanent state, redistribution of the energies occurs, which leads to a minimum energy density state. As shown in Fig. 2(a), the minimum energy density is non-negative up to a specific trackwidth and then becomes almost zero on increasing the trackwidth further. This variation can be explained by the competition between the exchange and the demagnetization energies, the dominant contributors to the total energy when an external voltage is absent. Exchange energy favours parallel alignment of the magnetization, whereas demagnetization energy favours the magnetic closure domains.^19,22^ As shown in Fig. 2(b), for elliptical rings with AR 1.3 : 1 up to 250 nm trackwidth, the required demagnetization energy to rotate the magnetization 180° is high. The initial energy density can not overcome it, which produces an onion state with transverse DWs having a non-negative minimum energy density. For trackwidths greater than 250 nm, required demagnetization energy to rotate the magnetization 180° is low, and the initial energy density can easily overcome it. This causes magnetization to flip over one-half of the ring, producing a vortex state with a negligible minimum energy density. Fig. 2(c) shows the corresponding simulated micromagnetics and PEEM images, where an onion state with transverse DWs up to 250 nm and a vortex state beyond 250 nm is obtained. The micromagnetic images are used to show the spin configuration of the rings whereas simulated PEEM images are used to display the evolution of magnetic domains.

It is to be noted that the application of the initialized external magnetic field along the major axis of the magnetic rings is not considered as the required external voltage to rotate DWs will be very high because of the high shape anisotropy energy of the elliptical ring.

### 3.2 Phase diagram

**Fig. 3:**
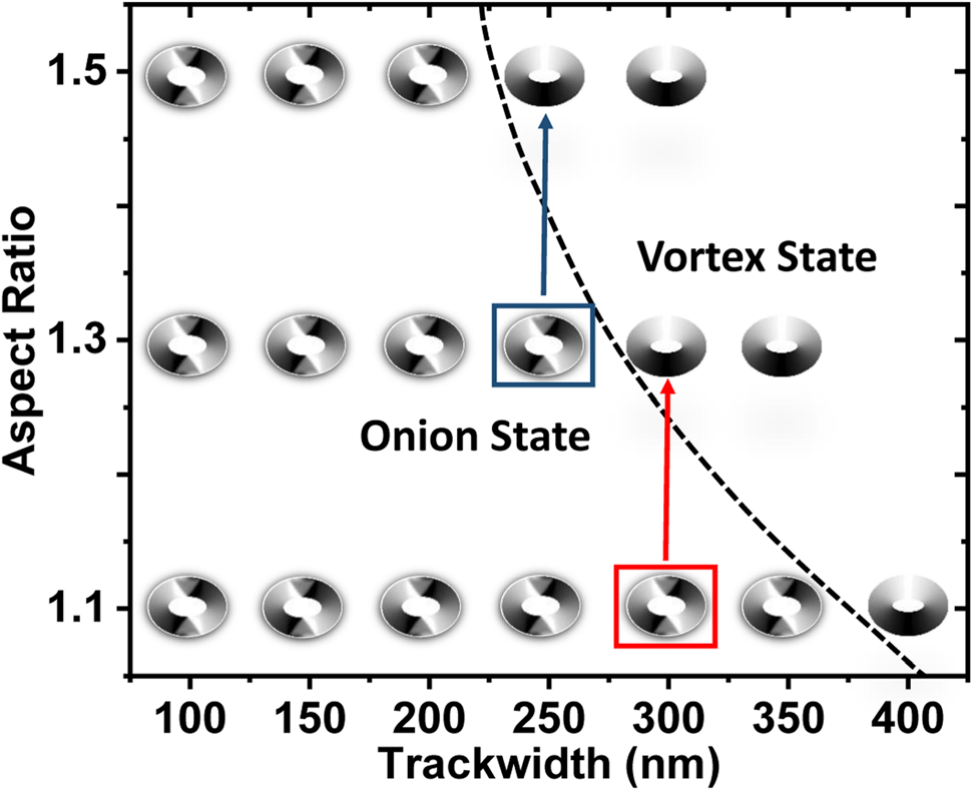
Phase diagram for simulated elliptical rings. (Black dotted curve: phase boundary between onion and vortex state, red and blue box: trackwidth corresponding to DW change from onion to vortex state when AR is increased from 1.1 to 1.3 and 1.3 to 1.5, respectively).

In order to predict the dependency of DW nature to the ring geometry, a phase diagram is plotted in Fig. 3 with varying AR and trackwidth for a fixed thickness. Trackwidth corresponding to the red (300 nm) and blue (250 nm) boxed area shows the changed nature of DW from onion to vortex when AR is increased from 1.1 : 1 to 1.3 : 1 and 1.3 : 1 to 1.5 : 1, respectively. At these particular trackwidths, the exchange energy starts to dominate compared to the demagnetization energy as the AR is increased. In Fig. 3, the black dotted curve shows the phase boundary between the onion and the vortex states for the geometrical variation of the magnetic ring. For further discussion, the maximum trackwidth which shows the onion state is represented by *t*_o_. *t*_o_ obtained for magnetic rings of AR 1.1 : 1, 1.3 : 1 and 1.5 : 1 has the values of 350 nm, 250 nm and 200 nm, respectively.

It is worth noting that only the onion state can couple to MNPs in a fluidic environment because of the finite value of the stray magnetic field.^9,10,19,24^ Thus in subsequent sections, only the onion state, *i.e. t* ≤ *t*_o_ for each elliptical ring is analyzed.

### 3.3 Domain wall rotation

After applying a voltage to the PMN-PT substrate, DW rotation is examined for each elliptical ring with the onion state (*t* ≤ *t*_o_). After initializing the onion state at pre-stressed condition, each magnetic ring is unstrained (*ε*_net_ = 0). The external voltage is applied and strain is instigated at the heterostructure interface due to the Villari effect.^22,23^ The generated strain modifies the stress anisotropic energy that competes with the initialized ring energies to bring it to the minimum energy state. A maximum applied voltage of 400 volts is considered as the IP anisotropic strain beyond this voltage is outside the linear piezoelectric response range of PMN-PT substrate and requires a phase transition.^19,35^ After ramping up the voltage from 0 volts to 400 volts with 40 V/ns ramp rate, two cases are observed depending on the trackwidth of the magnetic ring. Within the onion state, DW rotation up to 45° is observed up to a critical trackwidth represented by *t*_cr_, and complete 90° DW rotation is observed beyond *t*_cr_, as discussed next:

#### 3.3.1 In-plane DW rotation up to 45°

**Fig. 4:**
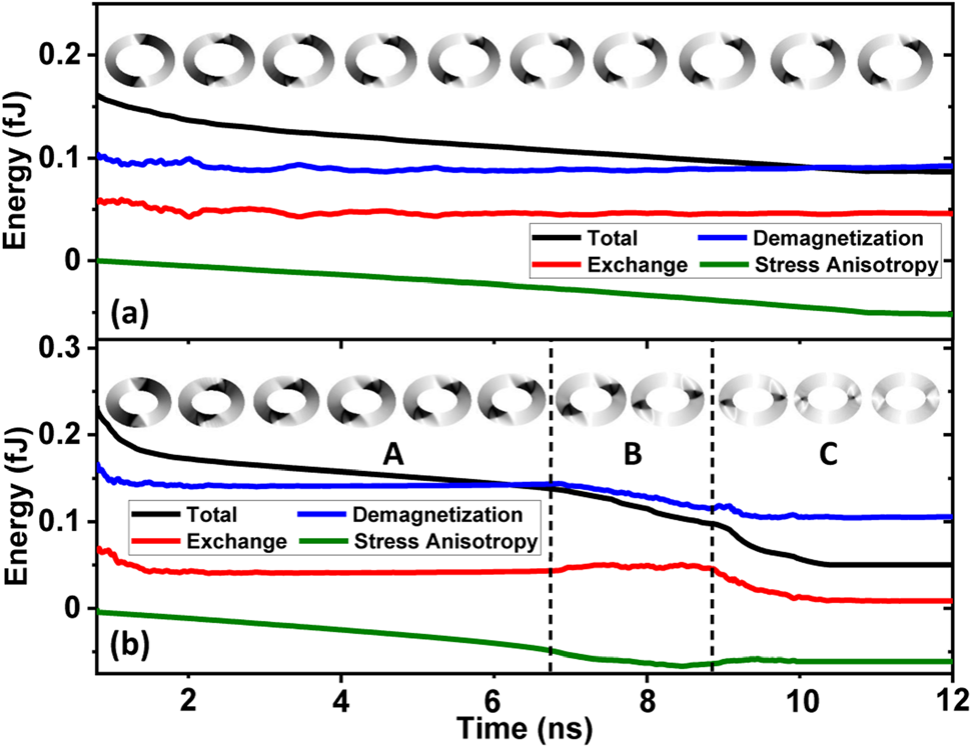
Total, exchange, demagnetization and stress anisotropy energy dynamics against time when an external voltage is ramped up from 0 volts. (a) For an elliptical ring with AR 1.3 and 150 nm trackwidth, total ring energy resulting from the stress anisotropy energy outweighs any substantial change to the initialized exchange and demagnetization energies. Simulated PEEM images shows initialized O–T to stable O–V transition and rotation towards tensile strain direction. (b) For an elliptical ring with AR 1.3 and 200 nm trackwidth, DWs start rotating toward the tensile strain direction and transform from an initialized O–T to an O–V state upon increasing the voltage (region A). Upon further increasing the voltage, stress anisotropic energy does not increase further and the total ring energy follows the demagnetization energy term and O–V DWs reorient towards the easy axis of the magnetic ring as shown in the simulated PEEM snapshots in region B. Finally, total ring energy starts following the exchange energy term that favours parallel alignment of the magnetization and the change in stress anisotropy and demagnetization energies remains almost negligible. DW again transforms from an intermediate O–V to final O–T state as shown in the simulated PEEM snapshots in region C.

**Fig. 5:**
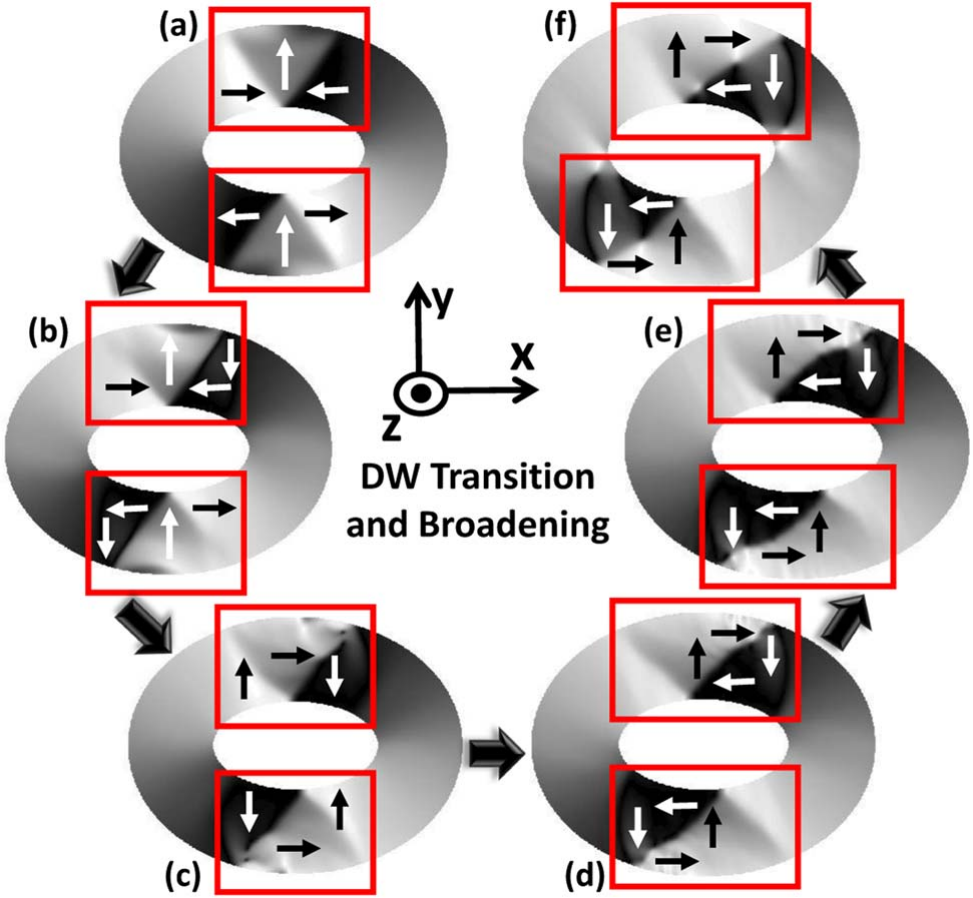
DW transition from onion state having transverse (O–T) DWs to onion state having vortex (O–V) DWs when an external voltage is ramped up from 0 volts. (a) For an elliptical ring with AR 1.3 : 1 and 250 nm trackwidth simulated PEEM image shows initialized O–T DW in boxed area at 0 volts. (b) and (c) Shows intermediate DW states once an external voltage is applied. As the initialized onion state is metastable, associated ring energies at applied voltage compete to bring magnetic ring into the lowest possible energy state and finally (d) O–V DW is observed. (c)–(f) Shows O–V DW broadening because of an elastic force generated due to stress anisotropy energy.

**Fig. 6:**
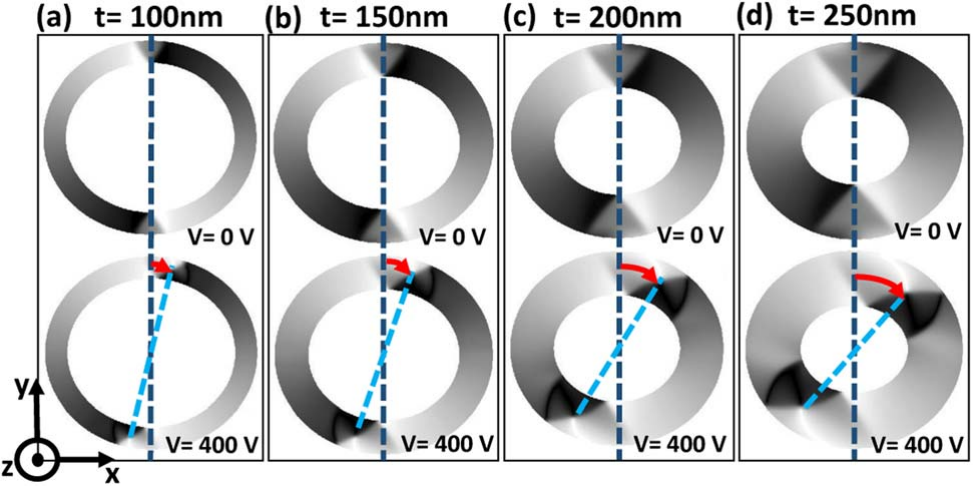
Increase in the reorientation angle towards tensile strain direction with increase in trackwidth. (a)–(d) Shows simulated PEEM image for an elliptical ring with AR 1.1 : 1 with increase in trackwidth from 100 nm to 250 nm in 50 nm steps. Blue dotted reference line indicates the initial DW position at a remanent state. Sky-blue dotted line indicates the reorientation of DW from the initial state with red curved arrow indicating the reorientation angle towards tensile strain direction at an external voltage of 400 volts.

For each magnetic ring up to a critical trackwidth (*t*_cr_), the generated strain introduces stress anisotropy energy that competes with the initialized ring energies. It is observed that the change in total ring energy resulting from the stress anisotropy energy outweighs any substantial change to the initialized exchange and demagnetization energies during DW rotation, as shown in Fig. 4(a). As a result, the DWs reorient toward a new easy axis created along the tensile strain direction as FeGaB is a positive magnetostrictive material.^46^ The rotated DWs also transitions from the initialized onion state with transverse (O–T) DWs to the onion state having vortex (O–V) DWs, as shown in Fig. 5(a–d). Such transitions occur because generated stress anisotropy energy modifies the DW magnetization configuration to move it to a more stable state.^19,53^ This signifies that O–V DWs are stable than O–T DWs when stress anisotropy is the dominant contributor to the total energy. Because of the simulation limit, the exact voltage where O–T to O–V transition occurs can not be determined. As shown in Fig. 5(d–f), DW broadening is also observed when voltage is ramped up as an elastic force is generated due to the stress anisotropy energy. Although it is possible that DW broadening can transform the DW from onion to vortex state completely,^53^ it is not observed in our model even after applying the maximum voltage. As shown in Fig. 6, increase in the reorientation angle towards tensile strain direction is observed with increase in trackwidth when a maximum voltage of *V* = 400 volts is applied. Consequently, maximum rotation (*δ*_max_) of such stable O–V DWs toward the tensile strain direction is observed at *t*_cr_. The observed *t*_cr_ for magnetic rings of AR 1.1 : 1, 1.3 : 1 and 1.5 : 1 are 300 nm, 150 nm and 50 nm, respectively and the corresponding *δ*_max_ are approximately 41.5°, 27.2° and 13.7°, respectively, being less than the ideal value of 45° relative to the initialized onion state. It is clear that *t*_cr_ and corresponding *δ*_max_ reduces with increase in AR.

#### 3.3.2 In-plane 90° DW rotation

As the trackwidth increases beyond *t*_cr_, complete IP 90° rotation is observed. Similar to case (3.3.1), DWs start rotating toward the tensile strain direction and transform from an initialized O–T to an O–V state upon increasing the voltage. The O–V DWs rotate up to 45° angle relative to the initialized state as total ring energy follows the stress anisotropy energy term, and the change in exchange and demagnetization energies remains almost negligible, as shown in region A of Fig. 4(b). Interestingly, upon further increasing the voltage, stress anisotropic energy does not increase further and the total ring energy follows the demagnetization energy term, as shown in region B of Fig. 4(b). Consequently, DWs reorient towards the easy axis of the magnetic ring as shown in the simulated PEEM snapshots in region B of Fig. 4(b). This signifies that the maximum stress anisotropic energy generated by the magnetic ring is limited^19^ and the potential *δ*_max_ possible due to that is 45° only. This signifies that additional rotation beyond 45° is not attributed to stress anisotropy energy. Instead, DW rotates beyond 45° as the shape anisotropy energy that originates from the demagnetization energy^54^ becomes the dominant contribution to the total energy. Consequently, a favourable energy term is created along the easy axis of the magnetic ring, and a complete IP 90° rotation is observed, as shown in region C of Fig. 4(b). Moreover, during this reorientation process, the rotated DW again transitions to the initial O–T state from the intermediate O–V state. Such transition occurs as soon as total ring energy starts following the exchange energy term that favours parallel alignment of the magnetization,^19^ and the change in stress anisotropy and demagnetization energies remains almost negligible, as shown in the simulated PEEM snapshots in region C of Fig. 4(b). This result is critical as the additional rotation of the DWs beyond IP 45° requires strains in multiple angles which is conventionally achieved by using a multi-electrode system^34–36^ as the effective stress anisotropic field described in eqn (7) is maximum at ±45° (or ±135°) and starts reducing after that.^55^ As the proposed model employs elliptical magnetic rings, additional rotation of the DWs beyond IP 45° is obtained by utilizing the shape anisotropy of the magnetic ring.

### 3.4 Domain wall reversibility

**Fig. 7:**
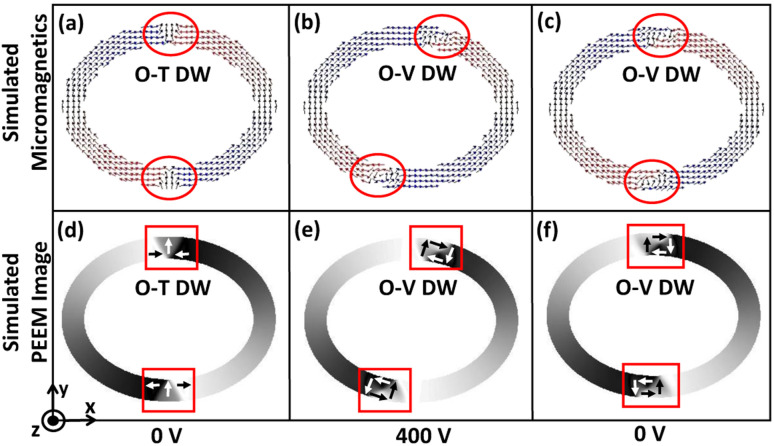
DW rotation towards tensile strain direction and simulated reversibility up to *t*_cr_. (a) Simulated micromagnetics for an elliptical ring with AR 1.3 : 1 and 150 nm trackwidth illustrates encircled initialized O–T DW at 0 volts. When an external voltage is ramped up from 0 volts transition from O–T DW to O–V DW occurs and (b) at 400 volts maximum rotation towards tensile strain direction is observed having O–V DW because of the dominant stress anisotropy energy. (c) Finally an external voltage is ramped down to 0 volts and complete reversal to the initial state is observed having 0 V DW. The O–V DW nature suggests the presence of an elastic force *i.e.* remanent strain because the nature of initial unstrained DWs is O–T. (d)–(f) Corresponding simulated PEEM images with boxed area illustrating type and position of DW.

The DW reversibility is also investigated after removing the external voltage. The external voltage is ramped down to 0 from 400 volts with a 40 V/ns ramp rate. This gives a different final DW state depending on the trackwidth of the magnetic ring. Up to *t*_cr_, we observe stable O–V DWs return to the initial position, as shown in Fig. 7(c and f). A complete reversal is observed because with ramping down of voltage, the associated stress anisotropy energy reduces at the same rate. As stress anisotropy energy is dominant in this case, its reduction imparts a driving force towards the initial unstrained state. However, the nature of rotated DWs remains O–V even after the complete removal of the external voltage, as shown in Fig. 7(c and f).

**Fig. 8:**
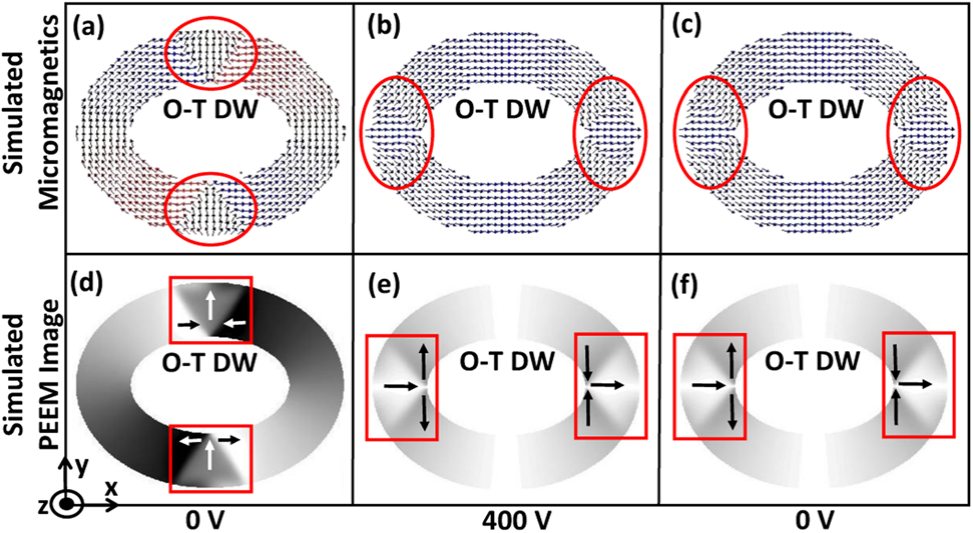
Complete IP 90° rotation and no reversibility for trackwidth greater than *t*_cr_. (a) Simulated micromagnetics for an elliptical ring with AR 1.3 : 1 and 200 nm trackwidth illustrates encircled initialized O–T DW at 0 volts. In this case intermediate O–V DW is metastable and again transforms to O–T due to energy minimization once voltage is increased. (b) At 400 volts complete IP 90° rotation is observed and (c) no reversibility is observed once an external voltage is ramped down to 0 volts again as shape anisotropy energy is dominant contribution of total energy and creates a favourable energy term along the easy axis of the magnetic ring. (d)–(f) Corresponding simulated PEEM images with boxed area illustrating type and position of DW.

On the other hand, no reversibility is observed when the trackwidth increases beyond *t*_cr_ as shape anisotropy energy is dominant in this case, as shown in Fig. 8(c and f). With reduction in voltage, even if the stress anisotropy energy imparts a driving force towards the initial state, its impact will be negligible. Also, the nature of DW remains same as the initial unstrained state. However, DW broadening is observed when the voltage is removed. This broadening signifies the presence of a remanent strain.^19^Table 2 shows a summary of the results obtained.

**Table 2:** DW rotation and reversibility for different AR elliptical rings

Trackwidth (nm)	AR 1.1 : 1	AR 1.3 : 1	AR 1.5 : 1
50	DW rotation towards tensile strain direction and reversibility possible	DW rotation towards tensile strain direction and reversibility possible	DW rotation towards tensile strain direction and reversibility possible
100	DW rotation towards tensile strain direction and reversibility possible	DW rotation towards tensile strain direction and reversibility possible	DW rotates 90° and no reversibility
150	DW rotation towards tensile strain direction and reversibility possible	DW rotation towards tensile strain direction and reversibility possible	DW rotates 90° and no reversibility
200	DW rotation towards tensile strain direction and reversibility possible	DW rotates 90° and no reversibility	DW rotates 90° and no reversibility
250	DW rotation towards tensile strain direction and reversibility possible	DW rotates 90° and no reversibility	Initial vortex state
300	DW rotation towards tensile strain direction and reversibility possible	Initial vortex state	Initial vortex state
350	DW rotates 90° and no reversibility	Initial vortex state	—
400	Initial vortex state	—	—

### 3.5 Magnetic nanoparticle-domain wall interaction

Based on the size-dependent DW rotation capabilities achieved for different magnetic rings, MNPs rotation is predicted. For this, the interaction of the DW and the injected MNP is analyzed first. As described in Section 2.2, force due to the stray magnetic field (*H⃑*_stray_) is the dominant contributor to total magnetic force generated by a magnetic ring, *H⃑*_stray_ for each magnetic ring is calculated to quantify the magnetic force. Initially, an external magnetic field is applied along the minor axis of the ring to reach magnetic saturation. Consequently, the magnetic flux around the ring is given by

16 *B⃑* = *μ*_0_(*H⃑*_external_ + *H⃑*_stray_

where *H⃑*_external_ is an external applied magnetic field and *H⃑*_stray_ is the stray magnetic field. *H⃑*_stray_ depends upon the material and shape of the magnetic element.^24,56^ After magnetic saturation is reached, the external magnetic field is removed (*i.e. H⃑*_external_ = 0). At the remanent state, new domains nucleate because of the energy minimization, giving rise to only *H⃑*_stray_. This modifies magnetic flux around the ring to

17 *B⃑*_stray_ = *μ*_0_*H⃑*_stray_

**Fig. 9:**
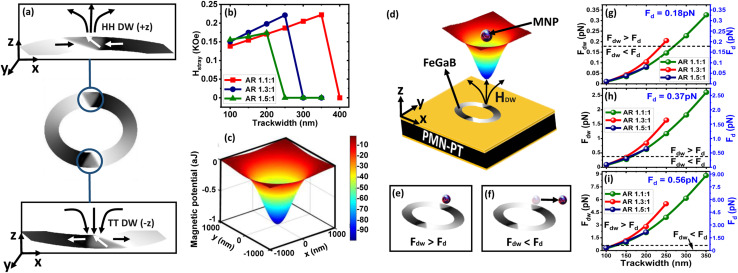
Generation of the stray magnetic field (*H⃑*_stray_) with radial distribution near domain wall (DW) junction. (a) Head to head (HH) DW initiates a stray magnetic field having a positive out of a plane component. Tail to tail (TT) DW initiates a stray magnetic field with a negative out of a plane component. (b) Stray magnetic field calculated for elliptical rings with AR 1.1 : 1, 1.3 : 1 and 1.5 : 1 as a function of trackwidth. For wider trackwidth magnetic ring (*t* > *t*_o_) magnitude of the stray magnetic field (*H⃑*_stray_ = *H⃑*_vortex_ ≈ 0) is almost negligible. For narrow trackwidth magnetic ring (*t* ≤ *t*_o_) magnitude of the stray magnetic is finite (*H⃑*_stray_ = *H⃑*_dw_ ≠ 0), giving rise to an onion state. (c) Generation of the attractive magnetostatic potential well between a 100 nm radius MNP and the center of HH DW (*x* = *y* = 0 nm) in an elliptical ring with AR 1.3 : 1 and 150 nm trackwidth. (d) Schematic of trapping of MNP due to an attractive magnetostatic potential energy well generated due to stray magnetic field (*H⃑*_dw_). (e) Coupling of the MNP to HH DW when *F⃑*_dw_ > *F⃑*_d_ and (f) decoupling of the MNP from DW when *F⃑*_d_ overcomes *F⃑*_dw_*i.e. F⃑*_dw_ < *F⃑*_d_. (g)–(i) Left ordinate shows attractive force (*F⃑*_dw_) calculated between (g) 100 nm (h) 200 nm and (i) 300 nm radius (*r*_p_) MNP at 200 nm height and HH DW in an elliptical ring with AR 1.1 : 1, 1.3 : 1 and 1.5 : 1 having *t* ≤ *t*_o_. Right side ordinate shows drag force (*F⃑*_d_) calculated for (g) 100 nm (h) 200 nm and (i) 300 nm radius (*r*_p_) MNP for injected velocity (*v⃑*_p_) of 0.1 mm s^−1^.

The source of generated stray magnetic field is demagnetization field.^17,57,58^ Using eqn (3) and (4), *H⃑*_stray_ is calculated as shown in Fig. 9(b). It is clear from Fig. 9(b) that for wider trackwidth magnetic ring (*t* > *t*_o_) exhibiting vortex state, magnitude of the stray magnetic field (*H⃑*_stray_ = *H⃑*_vortex_ ≈ 0) is almost negligible. Contrastingly, for narrow trackwidth magnetic ring (*t* ≤ *t*_o_) exhibiting onion state, the stray magnetic is finite (*H⃑*_stray_ = *H⃑*_dw_ ≠ 0). Since for a vortex state *H⃑*_stray_ ≈ 0, MNPs can not couple to a vortex state. In contrast, the onion state can couple to MNPs because of finite value of *H⃑*_stray_. The onion state separates two oppositely aligned magnetization regions or spin blocks (1) Head to Head (HH) and (2) Tail to Tail (TT) DWs. The peculiarity of these spin blocks is that they act as a magnetic pole where HH and TT DWs represent the north and south poles respectively.^17,58–60^ Consequently, these DWs initiate an inhomogeneous stray magnetic field with radial distribution, as shown in Fig. 9(a). For a HH DW, the direction of the stray magnetic field is outward, having a positive out-of-plane component. On the other hand, a TT DW creates a stray magnetic field directed inwards with a negative out-of-plane component. The stray magnetic field (*H⃑*_dw_) generated by HH DW creates an attractive potential well with magnetostatic energy (*E*) given by

18 *E* = −*μ*_o_*μH⃑*_dw_

where *μ*_o_, *μ* and *H⃑*_dw_ are free space permeability, magnetic moment of a nanoparticle and stray magnetic field generated by the ring respectively. Because commercial MNPs are superparamagnetic, it is assumed that *H⃑*_dw_ can not saturate these nanoparticles *i.e.*

19 *μ*_o_ = χ*H⃑*_dw_

where *χ* is the magnetic susceptibility of a nanoparticle. This modifies eqn (18) to20
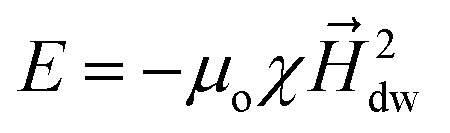


From eqn (20), it is clear that *E* depends on 
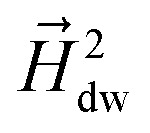
. As Fig. 9(b) shows the relation between *H⃑*_dw_ and trackwidth, a direct relation between trackwidth and magnetostatic energy (*E*) can be made, which indicates that *E* changes with trackwidth in the same manner as *H⃑*_dw_ changes with trackwidth.

Next, based on the above analysis, the MNP capture using DW is predicted. MNPs are captured when *H⃑*_dw_ prompts a magnetic moment in MNPs. This creates an attractive potential well with magnetostatic energy (*E*) localized at the center of HH DW, as shown in Fig. 9(c) and (d). Throughout our model, we assume that the center of the MNP is in close proximity to the magnetic ring with a constant height of 200 nm. As a result, force due to DW stray field attracts MNP towards HH DW. The value of this attractive force (*F⃑*_dw_) is given by eqn (11). As the onion state is observed for *t* ≤ *t*_o_, *F⃑*_dw_ for different AR elliptical rings having *t* ≤ *t*_o_ for a range of nanoparticle radius (*r*_p_) is calculated, as shown in left side ordinate of Fig. 9(g–i). As the initialized *H⃑*_dw_ is higher for intermediate AR (1.3) as compared to magnetic rings with AR 1.1 and 1.5, it is observed that *F⃑*_dw_ is larger for intermediate AR (1.3) for a fixed radius of MNP as the trackwidth increases. It is clear that, for a fixed radius of MNP and AR of an elliptical ring, the value of *F⃑*_dw_ is larger as the trackwidth increases. This is because the magnetoelastic energy (*E*) is significant for larger trackwidth in an onion state. This indicates that the strength of DW-MNP interaction for *t* ≤ *t*_o_ can be increased by making trackwidth larger. Although it may be possible that the presence of MNPs can distort the nature of DW,^61^ our model does not include such effect. As *F⃑*_dw_ directly depends upon nanoparticle volume 
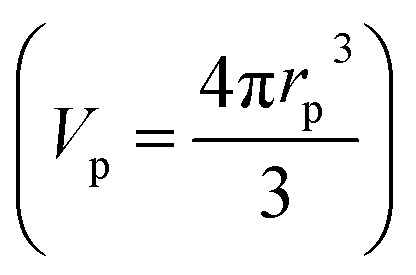
, *F⃑*_dw_ also increases with an increase in *r*_p_.

As mentioned in Section 2.2, as long as *F⃑*_dw_ surpasses *F⃑*_d_ (|*F⃑*_dw_| > |*F⃑*_d_|), nanoparticles bind to the HH DW, as shown in Fig. 9(e). On the contrary, if *F⃑*_d_ overcomes *F⃑*_dw_, the nanoparticle does not bind to the HH DW (Fig. 9(f)). As MNPs are injected with average velocity (*v⃑*_p_) of 0.1 mm s^−1^, next *F⃑*_dw_ is compared with the drag force (*F⃑*_d_). From eqn (14)*F⃑*_d_ calculated is 0.188 pN, 0.377 pN and 0.565 pN for *r*_p_ = 100 nm, 200 nm and 300 nm respectively as shown in right side ordinate of Fig. 9(g–i). Table 3 summarizes the comparison of *F⃑*_dw_ with *F⃑*_d_.Table 3 shows that the capture probability becomes more significant for bigger MNPs because of larger *F⃑*_dw_ than *F⃑*_d_. This indicates that the injected average velocity (*v⃑*_p_) should be lower to enhance the capture probability of smaller MNPs provided *r*_p_ ≥ *r*_c_.

**Table 3:** Comparison of *F⃑*_dw_ (pN) with *F⃑*_d_ (pN) for *r*_p_ = 100 nm, 200 nm and 300 nm respectively in an elliptical ring with AR 1.1 : 1, 1.3 : 1 and 1.5 : 1 at injected MNP average velocity (*v⃑*_p_) of 0.1 mm s^−1^. [Note: * trackwidth corresponding to *F⃑*_dw_ < *F⃑*_d_ and † trackwidth corresponding to *F⃑*_dw_ > *F⃑*_d_]

Trackwidth (nm)	*r*_p_ = 100 nm (*F⃑*_d_ = 0.1884 pN)			*r*_p_ = 200 nm (*F⃑*_d_ = 0.3768 pN)			*r*_p_ = 300 nm (*F⃑*_d_ = 0.5652 pN)		
	AR 1.1 : 1	AR 1.3 : 1	AR 1.5 : 1	AR 1.1 : 1	AR 1.3 : 1	AR 1.5 : 1	AR 1.1 : 1	AR 1.3 : 1	AR 1.5 : 1
100	0.009^*^	0.01^*^	0.012^*^	0.073^*^	0.086^*^	0.093^*^	0.247^*^	0.290^*^	0.312^*^
150	0.034^*^	0.043^*^	0.038^*^	0.270^*^	0.348^*^	0.305^*^	0.920^†^	1.17^†^	1.030^†^
200	0.078^*^	0.104^*^	0.081^*^	0.622^†^	0.837^†^	0.645^†^	2.100^†^	2.820^†^	2.177^†^
250	0.146^*^	0.205^†^	—	1.170^†^	1.640^†^	—	3.900^†^	5.500^†^	—
300	0.228^†^	—	—	1.820^†^	—	—	6.150^†^	—	—
350	0.327^†^	—	—	2.610^†^	—	—	8.830^†^	—	—

### 3.6 Magnetic nanoparticle manipulation using domain wall rotation

**Fig. 10:**
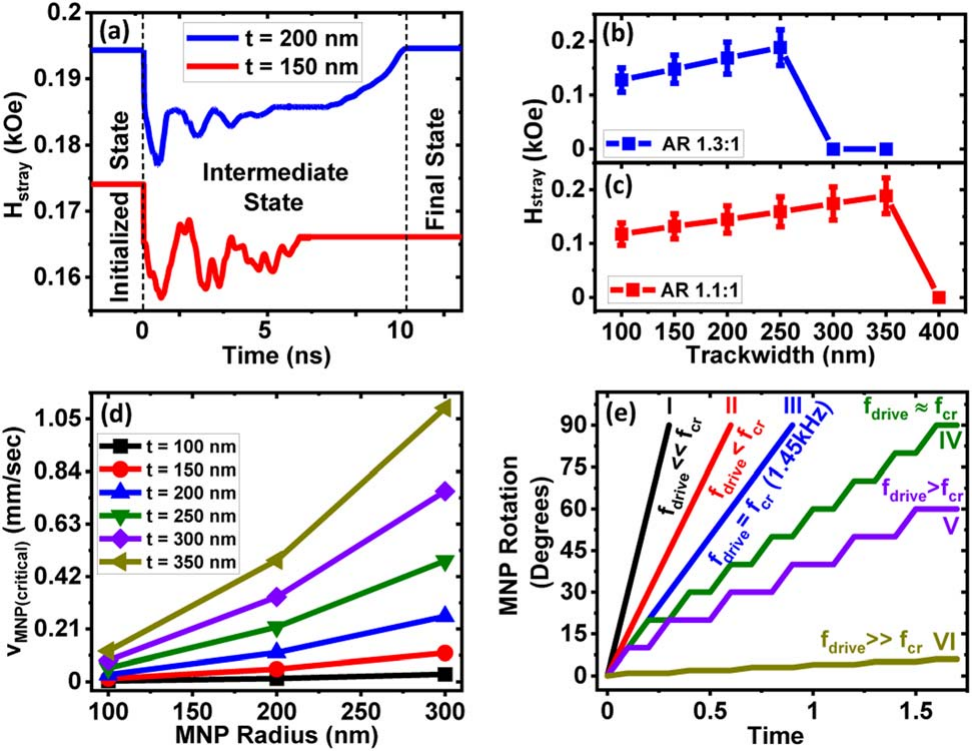
(a) Time-varying stray magnetic field profile for an elliptical ring with AR 1.3 : 1 and trackwidths 150 nm (*t* ≤ *t*_cr_) and 200 nm (*t* > *t*_cr_). Variation in the stray magnetic field for elliptical rings with (b) AR 1.3 : 1 and (c) 1.1 : 1 as a function of trackwidth. The error bar shows the maximum to minimum stray field variation, where the lower limit shows the minimum stray field possible during DW rotation by taking a conservative estimate of a 30% reduction in the stray field from peak value. (d) *v⃑*_(MNP(critical))_ profile for an elliptical ring with AR 1.1 : 1 and trackwidths 350 nm as a function of MNP radius. (e) MNP trajectories due to DW rotation as a function of *f*_drive_ for an elliptical ring with AR 1.1 : 1 and trackwidth 350 nm once an injected MNP of radius 300 nm is captured.

Once the injected MNP is captured in the magnetostatic potential energy well, MNP rotation after applying a voltage across the PMN-PT substrate is analyzed. Since the emanated stray magnetic field *H⃑*_dw_ from the DW could be different at different positions of the track due to DW transition (O–T to O–V or *vice versa*), a time-dependent stray magnetic field is calculated. The magnetization profile is utilized to calculate the stray magnetic field *via* the scalar magnetic potential using eqn (3) and (4). Based on the trackwidth of the magnetic ring, different stray magnetic field profiles are obtained. This difference is illustrated in Fig. 10(a), which shows a time-varying stray magnetic field profile for an elliptical ring with AR 1.3 : 1 and trackwidths 150 nm (*t* ≤ *t*_cr_) and 200 nm (*t* > *t*_cr_), respectively. As the voltage is applied at *T* = 0 s, a reduction in the stray field is observed from the initialized state. This reduction is observed since vortex walls exhibit a lower stray magnetic field as compared to the transverse walls^59,60^ and obtained DW starts transforming from an initialized O–T (Onion–Transverse) to an O–V (Onion–Vortex) state upon increasing the voltage, as shown in Fig. 5. For *t* ≤ *t*_cr_, as the voltage is ramped up further, the generated stress anisotropy energy modifies the DW configuration to move to a more stable state. As already discussed, O–V DWs are more stable than O–T DWs in this case; the lower stray field is observed in the final state. On the other hand, for *t* > *t*_cr_, as the voltage is ramped up from *T* = 0 s, DW initially transforms from an initialized O–T to an intermediate O–V state. Consequently, a lower oscillatory stray field is observed in the intermediate state. Since rotated DW again transitions to the initial O–T state from the intermediate O–V state in this case, the stray field starts increasing from the intermediate state, as shown in Fig. 10(a). This is observed because transverse walls exhibit a higher stray magnetic field as compared to the vortex walls.^59,60^ As a result, the final stray field is observed almost equal to the initialized state.

Since the continuous motion of the captured MNPs as a DW-MNP bound unit depends on the stray magnetic field that varies along the track, the minimum value of *H⃑*_dw_ from the intermediate state is considered for further analysis. We observe 5–20% reduction in the stray field from the initialized state to the minimum peak once the voltage is applied. We take a conservative estimate of a 30% reduction in the stray field. For instance, Fig. 10(b and c) shows variation in the stray magnetic field calculated for elliptical rings with AR 1.1 : 1 and 1.3 : 1 as a function of trackwidth. The error bar shows the maximum to minimum stray field variation, where the lower limit shows the minimum stray field possible during DW rotation. Using eqn (11), the critical force (*F⃑*_dw(critical)_) due to the minimum DW stray field is calculated. Next, using a model proposed by Bryan *et al.*,^62^ the critical MNP transport velocity (*v⃑*_(MNP(critical))_) is estimated by equating *F⃑*_dw(critical)_ with the viscous drag force (*F⃑*_d_) given by eqn (14). As shown in Fig. 10(d), *v⃑*_(MNP(critical))_ increases rapidly with MNP radius. The above analysis is crucial since for continuous MNP rotation along DW, the maximum DW speed (*v⃑*_dw_ = *v⃑*_dw(critical)_) should be less than or at most equal to the *v⃑*_(MNP(critical))_. This is why the minimum stray field is considered for the complete analysis.

Voltage driven DW speed (*v⃑*_dw_) is estimated using eqn (21) by geometrical consideration

21 *v⃑*_dw_ = *Cf*_drive_

where 
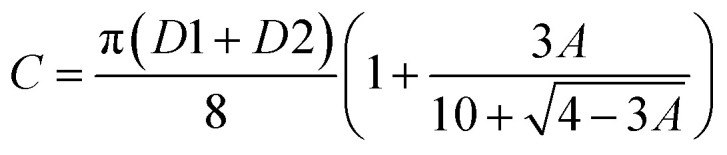
 represents quarter of the elliptical ring circumference with *D*1 and *D*2 as major and minor axis of the ring (Fig. 1(c)). *A* is given by22
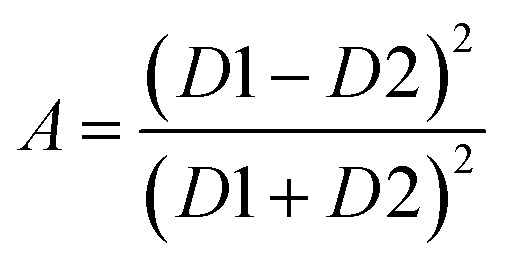


Quarter of the ring circumference is considered since maximum rotation possible is 90°. *f*_drive_ represents the voltage-induced DW rotation frequency once voltage is applied and given by23
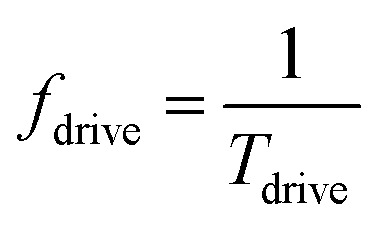
where *T*_drive_ represents the rate at which external voltage across PMN-PT is ramped up. Since simulations do not run in real-time, *f*_drive_ is analyzed analytically. Fig. 10(e) illustrates one such case where MNP trajectories due to DW rotation as a function of *f*_drive_ for an elliptical ring with AR 1.1 : 1 and trackwidth 350 nm is considered once an injected MNP of radius 300 nm is captured. Using Fig. 10(d)*v⃑*_(MNP(critical))_ is obtained as 1.09 mm s^−1^. Since for a continuous MNP rotation as a DW-MNP bound unit *v⃑*_dw_ should be less than or at most equal to the *v⃑*_(MNP(critical))_, using eqn (21)*f*_drive_ = *f*_critical_ is estimated considering *v⃑*_dw_ = *v⃑*_(MNP(critical))_. It is clear that for *f*_drive_ ≤ *f*_critical_ continuous motion of the captured MNPs as a DW-MNP bound unit will be observed, as shown in curves I, II and III of Fig. 10(e). On the other hand, if *f*_drive_ is made slightly higher than *f*_critical_ (*f*_drive_ ≈ *f*_critical_) a piecewise MNP rotation will be observed, as shown in curve IV of Fig. 10(e). This is because, in this case, MNP will be decoupled once DW starts rotating since *v⃑*_dw_ is slightly larger than *v⃑*_(MNP(critical))_ but immediately recoupled to a new DW position due to the generation of continuous DW attractive potential well train. This suggest a piecewise MNP rotation. As DW speed increases further, the probability of piecewise MNP rotation decreases, as shown in curve V of Fig. 10(e). For very large DW speed *i.e. f*_drive_ ≫ *f*_critical_, successful MNP rotation is not possible since *v⃑*_dw_ ≫ *v⃑*_(MNP(critical))_, as shown in curve VI of Fig. 10(e).

**Fig. 11:**
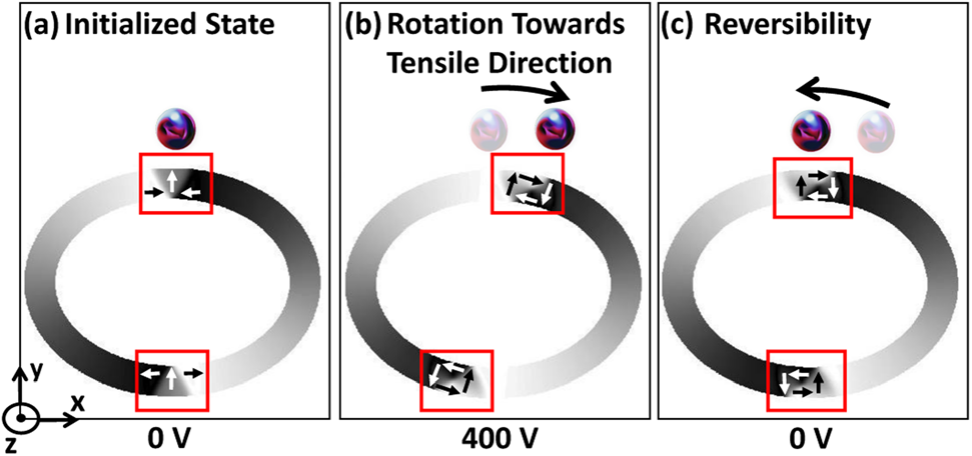
Trapping and manipulation of fluid borne MNPs up to *t*_cr_. (a) Trapping of MNP near initialized O–T DW at 0 volts for an elliptical ring with AR 1.3 : 1 and 150 nm trackwidth. (b) Maximum rotation towards tensile strain direction of trapped MNP at 400 volts and (c) MNP returns to the initial position once an external voltage is removed.

**Fig. 12:**
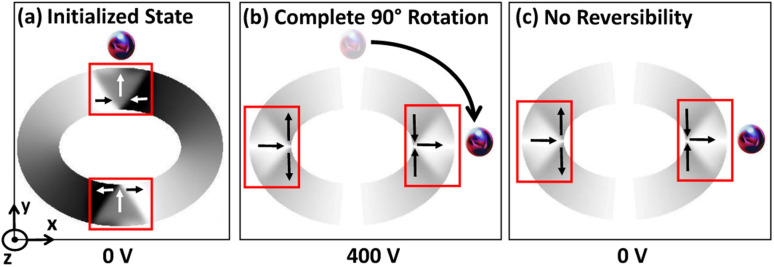
Trapping and manipulation of fluid borne MNP for trackwidth greater than *t*_cr_. (a) Trapping of MNP near initialized O–T DW at 0 volts for an elliptical ring with AR 1.3 : 1 and 200 nm trackwidth. (b) Complete IP 90° rotation of trapped MNP at 400 volts and (c) no reversibility of the trapped MNP once an external voltage is removed.

Once the MNP is trapped in the magnetostatic potential energy well and *f*_drive_ ≤ *f*_critical_, it follows the DW track along the circumference of an elliptical ring after applying a voltage across PMN-PT substrate. As previously mentioned, up to *t*_cr_, the DW reorientation angle increases with increase in trackwidth and maximum rotation (*δ*_max_) towards tensile strain direction occurs at *t*_cr_. Also, DW returns to the initial position upon removing an external voltage. As expected in this case, the reorientation angle of the captured MNP increases with increase in the trackwidth. At *t*_cr_, MNP can rotate maximum towards tensile strain direction and return to the initial position once an external voltage is removed, as shown in Fig. 11. On the other hand, beyond *t*_cr_, a favourable energy term along the easy axis of the magnetic ring is observed because of dominant shape anisotropy energy. Due to this, DW completes IP 90° rotation and does not return to the initial position upon removing external voltage. Consequently, captured MNPs complete IP 90° rotation with no reversibility, as shown in Fig. 12.

### 3.7 Estimation of the energy dissipation

Next, we estimated the energy dissipation by the magnetostrictive elliptical ring structure having a simple two-electrode system. We compared it with the magnetostrictive circular ring structure, which generally employs a multielectrode system with patterned top electrodes with the common ground for the additional rotation of the DWs beyond 45°.

#### 3.7.1 Energy dissipation for elliptical ring structure

The strain distribution in piezoelectric with fully covered top and bottom electrodes (two-electrode system) relies on piezoelectric coefficients.^63^ As already described in Section 3.3, we have considered a single crystal PMN-PT piezoelectric substrate with spontaneous polarization along 〈111〉 direction. With fully covered two electrodes, its 〈011〉 cut shows large IP anisotropic strain upon applying a voltage (V) with piezoelectric coefficients *d*_31_ and *d*_32_.^40^ Since outer diameter of the simulated elliptical ring along the major axis (*D*1) is 1 μm for all cases, 1.1 μm × 1.1 μm × 0.5 mm, substrate dimension is sufficient considering a single device operation. Once the voltage is applied across the piezoelectric, energy dissipation (*E*_d_) is given by24
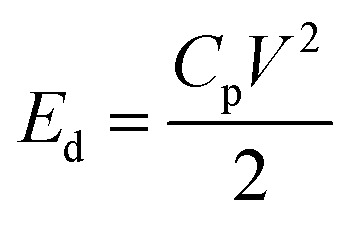
where *C*_p_ is the net piezoelectric capacitance. Since the dielectric permittivity (*ε*_R_) of *d* = 0.5 mm PMN-PT is in order of ≈1000, other line capacitances are neglected.^45^ Also, since internal dissipations due to Gilbert damping are negligible, these effects are also not considered. Thus, net piezoelectric capacitance will be25
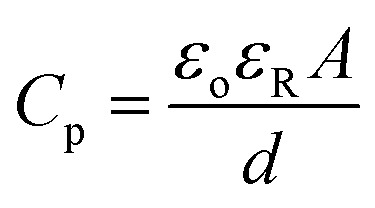
where *ε*_R_ is free space permittivity and *A* = 1.1 μm × 1.1 μm is cross-sectional area of PMN-PT. Thus, calculated maximum energy dissipation using eqn (24) is 1.7 pJ at 400 V.

#### 3.7.2 Energy dissipation for circular ring structure

Ideally, for a magnetostrictive circular ring, the maximum DW rotation possible due to a two-electrode system is 45°. Thus, a multielectrode system (patterned top electrodes with the common ground) is generally employed for the additional rotation of the DWs beyond 45°.^34–36^ In this case, pattern electrodes generate an axial strain of tensile nature along the line joining the electrodes upon applying a positive voltage.^31^ Note that since the poling direction of PMN-PT considered is 011 (+*z* direction), tensile strain is generated along the line joining the electrodes upon applying a positive voltage. For an opposite-poled PMN-PT, the nature of axial strain would be compressive for the positive applied voltage. We performed Finite Element Analysis (FEA) using COMSOL^64^ to obtain the strain profile of the PMN-PT substrate when patterned electrodes are used. To get maximum tensile strain, a maximum voltage of 400 V is given. It is observed that the minimum cross-sectional area of one electrode required is at least ≈660 nm × 660 nm to achieve the maximum axial tensile strain. The gap between the edges of the two electrodes is considered 1.01 μm, so a circular ring having the same dimensions (diameter = 1 μm) as that of an elliptical ring considered could be placed between the electrodes, although the gap required would be larger experimentally, which will reduce the axial strain.

**Fig. 13:**
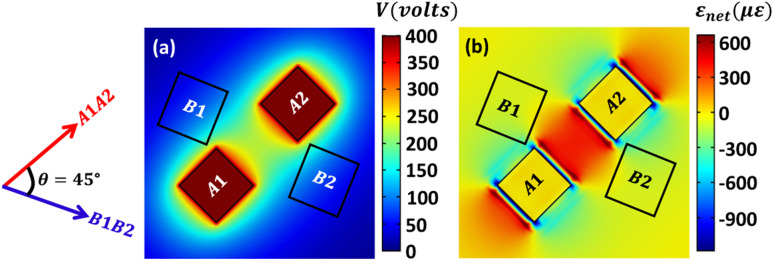
IP 90° rotation using a 4-electrode system. (a) Voltage profile once *A*1*A*2 electrode pair is given 400 V. (b) Corresponding net strain (*ε*_net_) profile of tensile nature along the line joining the electrode.

It is assumed that the initial DW position is 45° (anticlockwise) from the electrode pair *A*1*A*2. First *A*1*A*2 electrode pair is given 400 V, as shown in Fig. 13(a). Consequently, tensile strain (*ε*_[01−1]_) is generated along the line joining the electrode, as shown in Fig. 13(b). The value of compressive strain (*ε*_[100]_) is 0*με*. Although the generated strain is spatially varying, we have assumed its maximum value for simplification. At 400 V, a maximum net strain (*ε*_net_ ≈ 600*με*) of tensile nature is generated that approximately matches the tensile strain profile of the PMN-PT given in Fig. 1(b). Using eqn (25), the capacitance of single electrode *C*_*A*1_ = *C*_*A*2_ is 771*aF*. Since the applied voltage (400 V) and common ground are identical for both electrodes, *C*_*A*1_ and *C*_*A*2_ are parallel. Thus, net capacitance is *C*_*A*1_ + *C*_*A*2_ = 1542*aF*, when one electrode pair is activated. Therefore, the energy dissipated for a maximum 45° DW rotation is 1.2 pJ, based on eqn (24). For an additional DW rotation of 45°, again, 1.2 pJ energy will be dissipated once the *B*1*B*2 electrode pair is activated. Thus, the total energy dissipated by PMN-PT per 90° DW rotation is ideally 2.4 pJ. It should be noted that the generated maximum axial tensile strain (≈600*με*) is almost 40% compared to the maximum biaxial strain (≈1500*με*) generated due to fully covered top and bottom electrodes. Thus, a complete 90° DW rotation might not be possible using a four-electrode system, and additional electrode pairs would probably be required. Consequently, the actual energy dissipation could be more substantial. This clearly indicates that an elliptical ring can make particle manipulation more energy-efficient using a simple two-electrode system.

## 4 Conclusion

In conclusion, we have illustrated the remote and precise manipulation of MNPs in the fluidic environment. The manipulation mechanism relies on voltage-driven DW rotation in an elliptical-shaped ferromagnetic ring using strain-mediated MFs and their magnetostatic potential energy well trapping of fluid-borne MNPs. We observed DW transition from an initial metastable O–T state to a stable O–V state up to a critical trackwidth (*t*_cr_) when an external voltage is ramped up from 0 volts. These stable O–V DWs reorient towards the tensile strain direction after ramping the voltage further, and for *t* = *t*_cr_ the maximum reorientation towards tensile strain direction is observed. Finally, an external voltage is ramped down to 0 volts, and it is observed that stable O–V DWs return to the initial position. *t*_cr_ observed for magnetic rings of AR 1.1 : 1, 1.3 : 1 and 1.5 : 1 is 300 nm, 150 nm and 50 nm respectively, whereas corresponding *δ*_max_ are approximately 41.5°, 27.2° and 13.7°. On the other hand, we observed DW transition from an initial O–T state to an intermediate O–V state for trackwidth greater than *t*_cr_ when an external voltage is ramped up from 0 volts. The intermediate O–V DWs become metastable and convert to a stable O–T state after ramping up the voltage further, and complete IP 90° rotation is observed. Finally, an external voltage is ramped down to 0 volts, and it is observed that stable O–T DWs do not return to the initial position. This result is significant because the additional rotation of the DWs beyond IP 45° in a circular ferromagnet disc or ring using MFs requires strains in multiple angles, which is earlier achieved using a multielectrode system. As we have used an elliptical-shaped ferromagnetic ring, shape anisotropy creates a favourable energy term along the easy axis of the magnetic ring, and an additional rotation beyond IP 45° can be achieved for trackwidth greater than *t*_cr_ using two electrode system only. Using an analytical model, it is demonstrated that fluid-borne MNPs are magnetostatically coupled to DWs and for *f*_drive_ ≤ *f*_critical_ continuous MNP rotation is possible. It is observed that the capture probability becomes more significant for bigger MNPs, and to enhance the capture probability of smaller MNPs, injected average velocity should be lower. It is predicted that up to *t*_cr_, MNPs can rotate maximum IP 45° with *f*_drive_ ≤ *f*_critical_ and return to the initial position once an external voltage is removed. On the other hand, continuous IP 90° MNP rotation with no reversibility beyond *t*_cr_ is observed when *f*_drive_ ≤ *f*_critical_. Lastly, it is demonstrated that an elliptical ring can make particle manipulation more energy-efficient using a simple two-electrode system than a circular ring structure having multi-electrode system. The geometry-dependent manipulation capabilities of different AR elliptical-shaped ferromagnetic rings using strain-mediated MFs described above may be implemented for future energy-efficient compact lab-on-a-chip hyperthermia therapy applications. Also, the above work can be extended to manipulating biomolecules, proteins, DNA, cells *etc.*, which can be used for various medical and biological applications.

## Conflicts of interest

There are no conflicts to declare.

## Supplementary Material
